# 2.7 million samples genotyped for HLA by next generation sequencing: lessons learned

**DOI:** 10.1186/s12864-017-3575-z

**Published:** 2017-02-14

**Authors:** Gerhard Schöfl, Kathrin Lang, Philipp Quenzel, Irina Böhme, Jürgen Sauter, Jan A. Hofmann, Julia Pingel, Alexander H. Schmidt, Vinzenz Lange

**Affiliations:** 1 DKMS Life Science Lab, Blasewitzerstr. 43, 01307 Dresden, Germany; 2 DKMS, Kressbach 1, 72072 Tübingen, Germany

**Keywords:** Next generation sequencing, HLA genotyping, High resolution, High throughput, Amplicon PCR, DKMS, Primer dimers, PCR chimerism, Novel alleles

## Abstract

**Background:**

At the DKMS Life Science Lab, Next Generation Sequencing (NGS) has been used for ultra-high-volume high-resolution genotyping of HLA loci for the last three and a half years. Here, we report on our experiences in genotyping the HLA, CCR5, ABO, RHD and KIR genes using a direct amplicon sequencing approach on Illumina MiSeq and HiSeq 2500 instruments.

**Results:**

Between January 2013 and June 2016, 2,714,110 samples largely from German, Polish and UK-based potential stem cell donors have been processed. 98.9% of all alleles for the targeted HLA loci (HLA-A, -B, -C, -DRB1, -DQB1 and -DPB1) were typed at high resolution or better. Initially a simple three-step workflow based on nanofluidic chips in conjunction with 4-primer amplicon tagging was used. Over time, we found that this setup results in PCR artefacts such as primer dimers and PCR-mediated recombination, which may necessitate repeat typing. Split workflows for low- and high-DNA-concentration samples helped alleviate these problems and reduced average per-locus repeat rates from 3.1 to 1.3%. Further optimisations of the workflow included the use of phosphorothioate oligos to reduce primer degradation and primer dimer formation, and employing statistical models to predict read yield from initial template DNA concentration to avoid intermediate quantification of PCR products. Finally, despite the populations typed at DKMS Life Science Lab being relatively homogenous genetically, an analysis of 1.4 million donors processed between January 2015 and May 2016 led to the discovery of 1,919 distinct novel HLA alleles.

**Conclusions:**

Amplicon-based NGS HLA genotyping workflows have become the workhorse in high-volume tissue typing of registry donors. The optimisation of workflow practices over multiple years has led to insights and solutions that improve the efficiency and robustness of short amplicon based genotyping workflows.

**Electronic supplementary material:**

The online version of this article (doi:10.1186/s12864-017-3575-z) contains supplementary material, which is available to authorized users.

## Background

The hyperpolymorphic human leukocyte antigen (HLA) system, spanning about 4 Mb on the short arm of chromosome 6, contains a number of genes that play key roles in the adaptive immune response [1]. Especially the “classical” HLA genes encoding the 6 major antigen-presenting proteins (HLA-A, -B, -C, -DRB1, -DQB1, and -DPB1) play a crucial role in solid organ and haematopoietic stem-cell transplantation (HSCT), where outcome is mostly determined by the genetic concordance of HLA alleles between donors and recipients [2]. With more than 16,000 allelic variants identified today (http://www.ebi.ac.uk/ipd/imgt/hla/stats.html), combinatorial diversity in this region explodes, and the search for a matching unrelated donor can resemble the search for the proverbial needle in a haystack.

Despite 29 million potential unrelated donors for patients in need of an allogenic HSCT being currently registered worldwide (https://www.wmda.info), finding suitably matched donors can be severely hampered by the heterogeneous quality of the available genotyping information [3]. Until recently, unrelated stem-cell donor registries all over the world, which provide the bulk of this information, have utilised different serological and DNA-based HLA typing methods with variable resolution, such as sequence-specific oligonucleotide probes (SSOP), sequence-specific primer (SSP) PCR, or sequence-based typing (SBT) using Sanger sequencing.

However, these technologies are limited in throughput, precision and achievable coverage when compared to next generation sequencing (NGS)-based HLA typing methods [4]. Benefits of NGS-based typing approaches include high throughput through massive parallelisation, clonal sequencing of single molecules, sample multiplexing, and reduced costs per sample [5]. Whilst the extensive allelic diversity of HLA class I and II genes has made, and continues to make, high-resolution HLA typing challenging, the advances in NGS technologies have made ultra-high-throughput, cost-effective and precise HLA typing possible at an unprecedented scale [5–7].

**Fig. 1:**
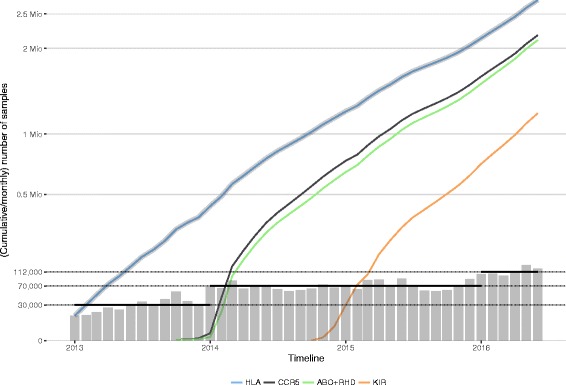
Cumulative and monthly numbers of donor samples genotyped at the DKMS Life Science Lab since 2013 as part of routine operations. The *grey line* shows the total cumulative number of genotyped samples, the *coloured lines* show gene-specific cumulative numbers; *grey-shaded bars* indicate monthly throughput. *Black horizontal bars* show (bi-)yearly mean throughput. The y-axis is square root scaled to enhance readability

To date, DKMS hosts HLA genotyping data for 7 million registered donors across Germany, Poland, the UK and the USA. Currently all DKMS samples from newly recruited donors are typed at the high-throughput genotyping facility DKMS Life Science Lab in Dresden, Germany. As of 2013, DKMS Life Science Lab successfully replaced a Sanger SBT workflow with a NGS HLA-typing workflow, initially based on Illumina MiSeq and later Illumina HiSeq 2500 amplicon sequencing [5]. Rapid advances in laboratory automation and increasing sequencing capacity have led to a dramatic growth from 30,000 donor samples processed every month to currently over 110,000 samples per month over the last three and a half years (Fig. 1). The total number of donor samples typed by NGS surpassed 2.7 million samples in June 2016 (Fig. 1), whilst the costs for HLA genotyping have dropped by more than 50% as compared to Sanger-based sequencing.

NGS technologies also make it easier to adapt read coverage to the experimental demand at minimal increases in cost. This results in an opportunity to expand the donor genotyping profile with ease and cost effectiveness by adding genes of interest that either may impact clinical outcome after HSCT (e.g., the KIR gene family), or that provide additional information to clinicians selecting the best possible donor (e.g., blood group markers, CCR5). Consequently, these markers were gradually added to the DKMS typing profile, starting with CCR5, ABO and RHD as of 2014 and followed by KIR genes as of 2015.

Using NGS technologies in a highly automated, high-volume production environment with high demands on data quality provides a number of key benefits over traditional Sanger sequencing and enables routine typing operations at an unprecedented scale. At the same time NGS poses a number of novel challenges and introduces complexities of its own. Here, we report on our experiences of using amplicon-based HLA typing by NGS at a massive scale. We present not only the performance metrics of our NGS-based typing approach but also key lessons we learned over a time period of three and a half years typing 2.7 million donors for six HLA loci.

## Results

### High throughput at high resolution

Between January 1, 2013 and June 30, 2016 a total of 2,714,110 samples were processed by amplicon-based NGS HLA typing first on the Illumina MiSeq platform until August 2014, and from then on predominantly on the HiSeq 2500 platform [5]. The move from MiSeq to HiSeq 2500 was driven by capacity demands and Illumina providing the “Rapid Run Mode” with 2×250 bp read lengths. The initially available read length of 2×125 bp on the HiSeq had not allowed for full coverage of the exons for our direct amplicon sequencing approach.

Since October 2013, 2,245,143 donors have additionally been typed for CCR5 and the blood groups ABO and RHD [8]; since October 2014 1,208,368 donors have additionally been typed for the presence/absence of KIR genes (Fig. 1).

The monthly throughput during the first year (2013) ranged from 14,862 to 56,493 (average 29,828) donor samples; this throughput then increased ranging from 57,294 to 90,316 (average 70,095) samples across 2014 and 2015, and increased further in 2016 ranging from 99,094 to 133,746 (average 112,358) samples (Fig. 1).

Based on data from the HLA core exons 2 and 3, between 96.78% (HLA-C) and 99.97% (HLA-DPB1) of the samples could be typed at high resolution or better as defined by EFI standard v6.3 (http://www.efiweb.eu/), with the exception that null alleles caused by a mutation outside of exons 2 and 3 remain unidentified (Table 1). For the remainder of the samples intermediate typing resolutions were obtained, with the exception of 21 low-resolution HLA-B samples (Table 1).

**Table 1:** NGS genotyping resolution for six HLA loci in 2.7 million DKMS donors

		Resolution		
Locus	Number of samples	High [%]^a^	Intermediate [%]	Low [%]
HLA-A	2,710,959	99.60	0.40	0
HLA-B	2,708,617	98.13	1.87	0.001
HLA-C	2,706,849	96.78	3.22	0
HLA-DRB1	2,710,549	99.92	0.08	0
HLA-DQB1	2,710,553	98.82	1.18	0
HLA-DPB1	2,706,356	99.97	0.03	0

^a^null alleles caused by a mutation outside of exons 2 and 3 remain unidentified

### Source material variability

For the vast majority (82%) of samples DNA is extracted from buccal cells. Donors are provided with two nylon DNA-sampling swabs (FLOQSwabs™ hDNA free, Copan Italia Spa, Brescia, Italy) and instructions to scrape the inside of the cheek with the swabs firmly for 30 s. These self-administered swabs are subsequently mailed to DKMS Life Science Lab for DNA extraction and genotyping.

**Fig. 2:**
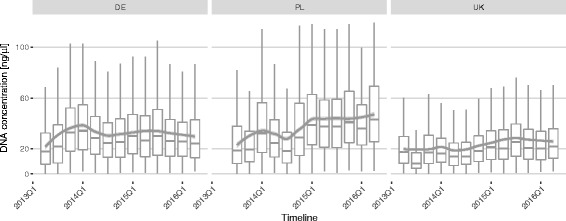
Quarterly average concentration of donor DNA extracted from buccal cells. Panels present differences between Germany (DE), Poland (PL) and the UK. Overall trend lines are generated by LOESS smoothing

DNA concentrations achieved by extracting from buccal samples have varied over a wide range. Ninety percent of all extractions yielded between 4.8 ng/μl and 86.1 ng/μl of DNA (median 26.6 ng/μl, *N* = 1,941,300). In addition, we observed marked fluctuations in median DNA concentrations over the complete time period and a strong dependence on sample provenance (Fig. 2). Buccal samples derived from UK donors generally yielded lower DNA concentrations (median 18.7 ng/μl [90% central range 3.9 to 62.7 ng/μl]) than samples derived from donors in Poland or Germany (median 33.5 and 26.5 ng/μl [90% central range 5.5 to 101.0 and 4.8 to 83.7 ng/μl], respectively). The reason for this discrepancy is unclear but might reflect differences in compliance, sample envelope material or sample transit time. No significant seasonal effects were detected.

It is possible that the large variability in the obtained DNA concentration is at least partially driven by differences in compliance with swabbing instructions by donors. For instance, a concerted effort in 2015Q1 by DKMS Polska emphasising the importance of the sampling procedure appears to have caused a dramatic increase in DNA yield from median 25.3 ng/μl (90% central range 3.6 to 92.6 ng/μl) before 2015 to 40.1 ng/μl (90% central range 8.5 to 105.1 ng/μl) in 2015 and 2016.

At least for Fluidigm-based workflows, DNA concentrations lower than 2 ng/μl have been found empirically to compromise genotyping results severely. Overall, 0.94% of all samples fell below this threshold, but the prevalence of such low-quality samples varied over time and with sample provenance (Additional file 1: Figure S1). These cases warrant a second extraction attempted using the alternative swab provided by the donor. Unfortunately, the first DNA extraction is a very good predictor of DNA concentrations obtained from the second swab (Pearson’s correlation, *r* = 0.79, *n* = 99,677, Additional file 2: Figure S2). Only 56.5% of the samples with an initial DNA concentration lower than 2 ng/μl achieved a DNA concentration higher than 2 ng/μl using the second swab. Since there was a 26-day period (90% central range 6 to 51 days) between the first and second extraction attempt, one may argue that the prolonged storage of swabs may have adversely affected DNA yield. However, samples with higher initial DNA concentration also tend to yield high concentrations in a second extraction (Additional file 2: Figure S2). This reinforces the notion that, to a large extent, individual patterns of compliance impact the yield from DNA extractions.

### Read quality

**Fig. 3:**
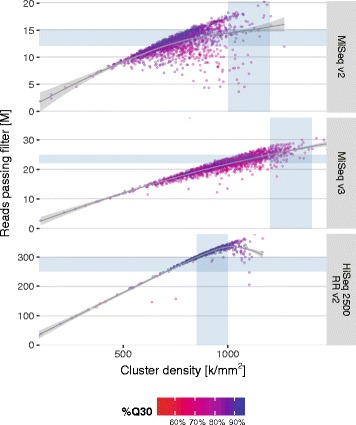
Reads passing filter vs. cluster density on Illumina MiSeq and HiSeq instruments. Each data point represents a run (flowcell). Shaded areas denote supported ranges of cluster densities and expected output for different chemistries/kits as specified by Illumina. The colour gradient indicates the total percentage of bases reaching a quality score of 30 or higher per run. Trend lines are generated by generalised additive model fits using a cubic penalised regression spline. M = millions

High-quality and unbiased sequence read data obtained from the sequencing platform greatly facilitate accurate and reproducible high-throughput typing. For Illumina instruments, the two common metrics for overall run performance are the number of reads passing filter (PF reads), i.e., reads that pass an internal quality filtering procedure (chastity filter), and the total percentage of bases that are assigned a Phred quality score of 30 (99.9% accuracy) or better (%Q30). The density of clonal clusters on Illumina flow cells is expected to strongly influence overall performance. By increasing the cluster density the read yield is increased until too many clusters are so close that they cannot be separated algorithmically. At this point, read yield saturates and may even decline and, according to Illumina documentation, sequencing quality may suffer. Illumina suggests optimal ranges of cluster densities and the corresponding expected outputs for different sequencing chemistries and instruments (compare Fig. 3). Achieving a designated cluster density requires loading the correct amount of high-quality library DNA onto the flow cells.

Special considerations, however, apply for sequencing from low-diversity libraries such as libraries generated from amplicons. Diverse or balanced libraries (e.g., libraries generated from random fragments) show an approximately equal distribution of all four nucleotides in every cycle. The Illumina RTA (Real Time Analyzer) software originally was optimised using balanced libraries to accurately locate cluster coordinates during the first sequencing cycles (template generation). Amplicon-based libraries, in contrast, tend to show a biased nucleotide distribution which may lead to a failure to segregate adjacent clusters and can adversely affect yield as well as data quality. Even though recent RTA versions have been optimised to be more robust with regard to low-complexity libraries, Illumina still recommends spiking in 5-10% PhiX for increasing diversity and targeting a more conservative cluster density.

For our NGS-based routine typing operations we have thus far performed a total of 3,642 runs distributed across two Illumina platforms (3,331 runs on MiSeq instruments, 311 runs on HiSeq 2500 instruments) and 3 versions of sequencing chemistry (MiSeq reagent kit v2, 1621 runs; MiSeq reagent kit v3, 1710 runs; and HiSeq Rapid Run SBS Kit v2, 311 runs). Whilst we initially tried to attain the cluster densities supported for balanced libraries it quickly became clear that our low-diversity HLA libraries required a reduction in template input for optimal yield and data quality (Fig. 3). This effect was especially noticeable early on for the MiSeq v2 chemistry where a number of runs showed reduced PF read counts at supported cluster densities or higher (Fig. 3, upper panel). With MiSeq v3, less data degradation was observed at the supported cluster densities but the strategy to undercluster was retained since optimal yield was readily achieved even at lower densities (Fig. 3, middle panel). HiSeq flow cells with Rapid Run SBS v2 chemistry could safely be clustered at the supported upper limit of 1,000 k/mm^2^ without sacrificing a linear increase in yield at all (Fig. 3, lower panel). Interestingly, no obvious relationship between achieved cluster densities and %Q30 became apparent in our runs. The most likely explanation for amplicon libraries performing markedly better on MiSeq v3 and especially the HiSeq is that new versions of the RTA software were rolled out by Illumina with algorithmic improvements to better cope with low-diversity libraries.

A critical factor influencing data quality and yield are template-independent primer-primer interactions that take place during the PCR steps and give rise to artificial products, especially primer dimers (PDs) [9]. Although primer dimer formation can be reduced by careful primer design and the application of stringent PCR conditions, it becomes increasingly difficult to avoid all primer interactions when developing multiplexed reactions. For the Fluidigm workflow, before the split of low-concentration and high-concentration samples into separate workflows, we experienced average monthly PD rates ranging from 34.0% ± 14.5% SD in January 2014 to 4.5% ± 1.9% SD in November 2015. Average monthly PD rates were independent of sequencing instrument and/or chemistry (two-way ANOVA, *F*_2,35_ = 1.76, *P* = 0.19) but decreased significantly over time due to continuous process optimisations and tweaking of the primers used in routine operations (two-way ANOVA, *F*_24,35_ = 46.3, *P* < 0.001, Additional file 3: Table S1).

A particularly troublesome source of increased PD formation was identified after a careful analysis of the sequences of primer dimer products. It showed that the primer sequences involved exhibited recurring patterns of degradation at their 3’-ends. We tracked the cause to the hot-start PCR system used (Roche FastStart High Fidelity PCR System). In contrast to the documentation’s claim of “inactivity at low temperatures”, only the polymerase activity is minimised at ambient temperatures by the hot-start modifications. The 3’-exonuclease providing the proofreading capabilities is not modified to require heat activation. As a consequence, the 3’-exonuclease degrades primers at the 3’-end in the reaction mix during cooled storage and reaction setup. A change in protocol was therefore applied during May/June 2015 where standard primers were replaced by modified primers incorporating three phosphorothioated nucleotides at the 3’-end to inhibit exonuclease degradation (PTO primers). Prior to this protocol change we observed an average PD rate of 17.0% (±8.6%, *n* = 59,534, April 2015). After the protocol change was fully implemented the average PD rate dropped to 6.4% (±2.6%, *n* = 62,695, July 2015).

The PD problem was further exacerbated by a peculiarity of our amplification protocol: In a standard PCR setup, PDs derived by 3’-end degradation are expected to form an inefficient substrate for amplification in subsequent PCR cycles as the majority of primers continue to carry intact 3’-ends and fail to bind to the degraded PDs. In contrast, with the 1-PCR 4-primer approach used in our Fluidigm workflow, we use two inner primers with target-specific 3’-tails and a 5’-tail complementary to two outer indexing primers [5]. Thus, PDs formed by the inner primers constitute an appropriate substrate for further amplification by the outer primers in subsequent cycles.

The notion that the 1-PCR 4-primer (Fluidigm) protocol increases the risk of PD formation was also supported by a significant reduction in PD rates after the alternative 2-PCR 2-primer (384 PCR) protocol was introduced for low-DNA-concentration samples in November 2015 (Fluidigm: 3.25% ± 0.98% PD rate; 384 PCR: 0.9% ± 0.3% PD rate; two-way ANOVA, *F*_1,13_ = 36.7, *P* < 0.001). The move from the 1-PCR 4-primer setup to the 2-PCR 2-primer setup is confounded by a commensurate move from the Fluidigm nanofluidics platform to standard 384-well plates with significantly larger reaction volumes and consequently different reaction kinetics. However, the KIR genes were always amplified on plates. To tease apart the relative contributions of PCR protocol and reaction volume, we analysed different HLA and KIR amplicons separately (Additional file 4: Figure S3). Disregarding amplicons with negligible PD rates (<0.1%) and an extreme outlier (HLA-A exon 2, Additional file 4: Figure S3), the remaining 6 HLA and 3 KIR amplicons had similar decreases in average PD rates when moving from a 1-PCR 4-primer approach to a 2-PCR 2-primer setup (-86.0% ± 19.5% for HLA, -86.6% ± 15.2% for KIR). This suggests that there is no correspondence between reaction volume and the degree of primer dimerisation.

**Fig. 4:**
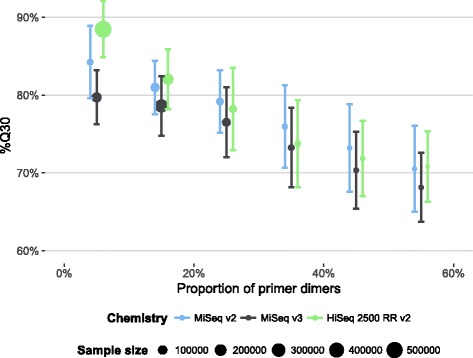
Average percentage of bases reaching a quality score of 30 or higher per run (± SD*)* vs. proportion of primer dimers (binned into 10% intervals)

High proportions of primer dimers not only reduce the number of reads on target per sample but also influence overall run quality by lowering %Q30 (Fig. 4, linear model, average reduction %Q30 = -3.4% ± 0.004% per 10% increase in PD rate, *t*_1452986_ = - 924.3, *P* < 0.001). This is probably explained by the fact that we are sequencing more cycles than the average length of the primer dimers whereby the polymerase runs off the template and produces low-quality data for the remaining sequencing cycles.

**Fig. 5:**
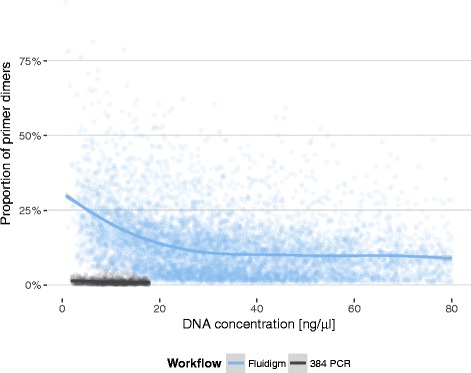
Dependency of primer dimer rate from initial DNA concentration and workflow. The two workflows differ both in their reaction volumes and amplification strategies (Fluidigm: single PCR, 4 primers; 384 PCR: 2 PCRs, 2 primers each). Solid lines depict generalised additive model fits using a cubic penalised regression spline. The shaded bands around the regression lines indicate the pointwise 95% confidence intervals on the fitted values

Independent of primer design and PCR conditions, we also found template DNA concentration to influence primer dimer rates within the nanofluidic Fluidigm workflow. At DNA concentrations below 30 ng/μl we observed a steep rise in the average PD rate, whereas the PD rate remained relatively constant at higher concentrations (Fig. 5). Whilst low target copy numbers have been reported before as favouring primer oligomerisation and mispriming [10], this effect appears to be exacerbated on a nanofluidics platform. When the 384 PCR workflow was introduced for low-concentration samples in November 2015, we observed an overall marked decrease in PD rate in this workflow (discussed above, Fig. 5). Independent of this effect, however, both the Fluidigm workflow (linear model, *t*_9998_ = -31.3, *P* < 0.001) and the 384 PCR workflow (linear model, *t*_9998_ = -15.4, *P* < 0.001) showed significant rates of increase in PDs with decreasing DNA concentrations (based on 10,000 samples drawn randomly from each workflow with DNA concentrations of less than 18 ng/μl). The magnitude of the effect was slightly, albeit significantly, larger in the Fluidigm workflow with an estimated 3.4% (95% CI = 3.2%–3.6%) change in PD rate for each ng/μl DNA less compared to the 384 PCR workflow with an estimated 2.7% (95% CI = 2.3%–3.0%) change in PD rate for every ng/μl DNA reduction.

We conclude that we were able to reduce the PD rate from 17% to less than 1% mainly by (a) using PTO-modified primers to avoid degradation and (b) moving from a 1-PCR 4-primer setup to a more conventional 2-PCR setup for target-specific amplification and barcoding PCR. Given the strong effect on overall run quality, monitoring and minimising PD rates seems highly advisable when performing amplicon-based NGS experiments.

### Read artefacts

In addition to primer dimer formation, amplicon-based sequencing is subject to another frequent methodological pitfall, namely the PCR-mediated formation of cross-over products between the two allelic variants, also called PCR chimerism [11, 12]. These reads incorporate portions of both alleles present in the sample into a single sequence, forming recombinants between the starting templates. Chimeras are more likely to be observed with increasing numbers of PCR cycles and if the initial concentration of templates is high [13]. Such cross-over events can be filtered by our analysis software as long as the parent sequences are not significantly outnumbered by the recombinant sequences. In addition, chimeras combining target and off-target reads stemming from, for instance, HLA-DRB3/4/5 or HLA-H are especially challenging to detect. High rates of chimeric reads can therefore confound the ability to successfully call the correct alleles [5].

**Fig. 6:**
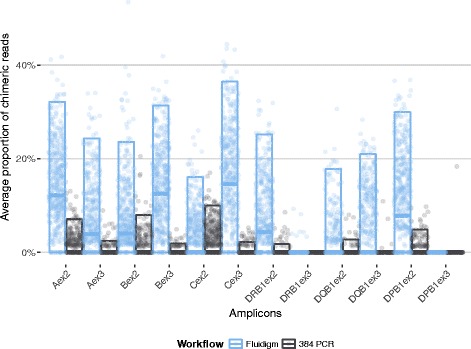
Proportion of PCR-mediated recombinant reads (chimeric reads) for different HLA amplicons and different workflows (see Methods for details)

The observed rate of chimeras depends heavily on the amplified locus and exon (Fig. 6; Fluidigm workflow, one-way ANOVA, *F*_11,3683_ = 82.5, *P* < 0.001; 384 PCR workflow, one-way ANOVA, *F*_11,1754_ = 55.2, *P* < 0.001). HLA-C exon 3 consistently shows the largest average proportion of chimeras amongst on-target reads (median 15.2%; IQR 4.6 to 23.6%), whilst HLA-DPB1 exon 3 and HLA-DRB1 exon 3 rarely show any sign of chimeric reads. The low chimerism rates observed for exon 3 of these class II HLA genes is possibly explained by the fact that the currently known exon 3 allelic sequences exhibit little variability, which facilitates escaping chimerism detection.

As with the other aforementioned metrics of quality, we observed a dramatic reduction in cross-over rates for the 384 PCR workflow (Fig. 6, linear model, average reduction in chimeric reads = -36.6% ± 1.2%, *t*_5437_ = -30.4, *P* < 0.001). This seems counterintuitive given that the Fluidigm workflow has fewer amplification cycles compared to the 384 PCR workflow (35 cycles and two steps of 35 and 10 cycles, respectively).

However, it has been observed that chimera formation largely occurs at late PCR cycles [14, 15] and is also promoted by higher initial template concentration [13]. In the Fluidigm PCR reactions initial template DNA concentrations are much higher (at least 8.8 ng/μl in an 11 nl reaction volume) than in the 384 PCR reactions (at most 2.7 ng/μl in a 10 μl reaction volume). This is to ensure that sufficient product for downstream processing is generated in the much smaller reaction volumes on Access Array chips. Unfortunately, as these reactions now reach saturation earlier they also offer more opportunities for crossing-over at earlier cycles [13].

### Quantification-free post-PCR normalisation

Ideally, each PCR product should be quantified individually and subsequently pooled equimolarly. However, to keep the workflow simple and cost-effective we opted to pool PCR products without attempting to normalise DNA concentrations.

**Fig. 7:**
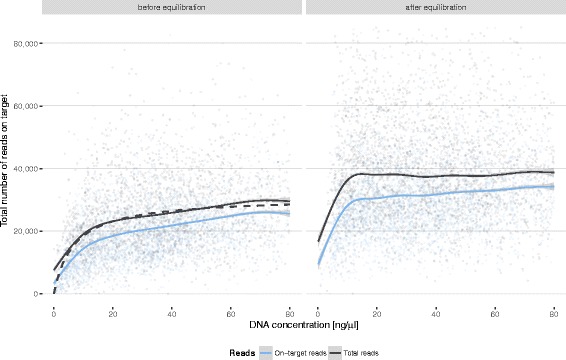
Correlation between initial DNA concentration and read coverage per sample before (left panel) and after (right panel) August 2014. In August 2014 a new post-PCR equilibration strategy was introduced based on a Michaelis-Menten saturation curve (dashed line, left panel) estimated from the data. Solid lines show generalised additive model fits using a cubic penalised regression spline. The shaded bands around the regression lines indicate the pointwise 95% confidence intervals on the fitted values

As data from samples amplified on Fluidigm Access Array chips accumulated, we found a weak but significant relationship between initial DNA concentration and the final number of reads generated for a sample (Fig. 7, left panel). Initial DNA concentration *C*_*i*_ and final number of reads per sample *R* show a saturation dependency that can be modelled by a Michaelis-Menten equation:

$$ R={C}_i/\left({C}_i+{K}_m\right){R}_{\max } $$

Fitting a two-parameter Michaelis-Menten model to the available data allowed for estimating the Michaelis-constant, *K*_*m*_, and the maximum achievable read number, *R*_max_, for our system. Thus, we could use the overall *K*_*m*_ estimate to predict relative final read counts for samples processed on an Access Array chip and adjust the amount of PCR product entering the post-PCR pool to minimise the effect of differences in DNA concentrations. Implementing this quantification-free post-PCR normalisation strategy balanced read coverage further across a wide range of initial DNA concentrations (Fig. 7, right panel). Only samples with DNA concentrations of less than 5 ng/μl yield too little PCR product to enable complete compensation.

### Read coverage

For reliable genotyping it is advantageous not only to achieve a balanced depth of coverage across samples but also across amplicons. Analysing 468,305 donor samples distributed over 299 MiSeq/HiSeq runs performed between January 2016 and June 2016, on average 56,200 (interquartile range [IQR] 44,560–69,690) reads were produced per sample. Of these reads, 95.8% (IQR 94.3–96.8%) mapped to their respective target amplicons and could be used for subsequent analyses. These reads partition into those targeting HLA loci (median 19,463; IQR 14,586–25,695), KIR loci (median 25,415; IQR 18,617–33,406), and others (CCR5 and blood groups ABO and RHD; median 6,127; IQR 4,178–9,943).

**Fig. 8:**
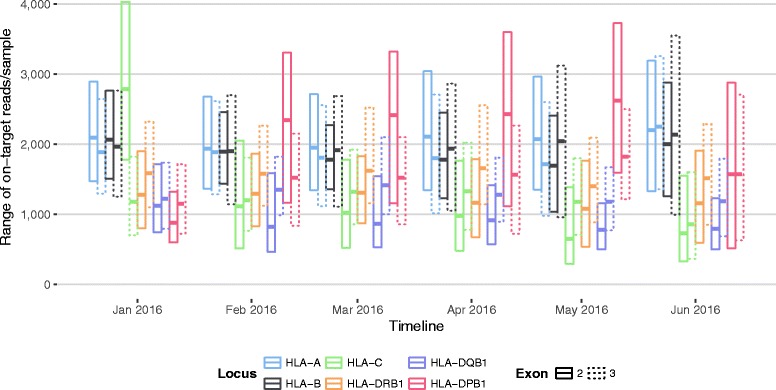
Distribution of on-target paired-end reads across amplicons. Different colours indicate different HLA loci. Solid lines indicate exon 2 amplicons; dashed lines indicate exon 3 amplicons

The number of on-target reads for individual HLA amplicons varied across loci and exons (Fig. 8; two-way ANOVA, *F*_11, 49726_ = 468.3, *P* < 0.001). Across amplicons we obtained a median coverage ranging from 867-fold (HLA-DQB1, exon 2) to 2034-fold (HLA-A, exon 2). However, the relative read counts across amplicons have also changed over time as primers were tweaked to try to equalise read counts as much as possible (two-way ANOVA, *F*_5, 49726_ = 13.1, *P* < 0.001). Uneven read depths across amplicons are due to different primer efficiencies and amplification biases where some sequences tend to amplify better than others [16]. Different primer efficiencies can be alleviated by tweaking either the primer sequence itself or by adjusting the relative amount of primer added to the PCR reaction, with the former being undertaken after the initial primer design. Unfortunately, it turns out that some degree of adjusting the relative amount of primer may become necessary when new batches of reagents are put into use (approximately once per month). However, despite perfectly adjusted primer sets, sample-to-sample variations (for example, in concentration and purity) resulted in diverging amplification kinetics and varying endpoints. Therefore, only post-PCR quantification and normalisation would deliver fully balanced amplicons. Costs for such a quantification step for each amplicon would increase current sequencing costs several fold. The low cost of sequencing allowed us to bypass such normalisation by way of massively oversampling for most samples and targets. Thereby, despite omitting quantification and normalisation, 98.6% of all HLA amplicons achieved more than 100x on-target coverage, which is sufficient for genotyping at the highest possible quality.

### Sample repetition

On occasion, the initial genotyping attempt fails to meet quality standards and the affected sample has to be reanalysed. This can affect either individual loci or the entire sample. A full sample repeat typing including a second DNA extraction is triggered if the initial DNA concentration is less than 2 ng/μl or more than 4 HLA loci fail to produce credible results.

We can identify several common factors that may trigger repeat typing of an individual locus: Technical concerns include too low total read numbers, too few reads on target, or heavy imbalances across alleles. Other causes for concern are the presence of more alleles than expected, which might be a result of one or more of many factors such as PCR chimerism, sample contamination, co-amplification of closely related loci, misamplification or sequencing errors. An analysis must also be repeated if the genotyping software fails to construct a genotype. This may occur for benign reasons (e.g., a novel allelic sequence is encountered) or be a result of stochastic allelic dropouts during PCR [17]. More insidiously, allelic dropouts may remain “silent”, i.e., be technically valid but erroneous homozygous or even heterozygous genotypes may arise [17]. Therefore, machine learning techniques are employed to help identify “implausible” genotypes that warrant repeated analyses to check their validity [17].

Whilst we strive to keep the rate of repetition low for economic reasons, it is important to employ conservative quality thresholds and repetition criteria to achieve the highest possible reliability of genotyping results. Repeat genotyping runs are automatically suggested by the genotyping software based on conservative thresholds for quality metrics and triggered manually after inspection of suspicious typing results [5]. In addition, analysts may trigger repeat typings for putative novel alleles and in cases where genotypes suggested by the software are considered to be implausible (e.g., genotypes that have rarely been encountered before in our sample populations).

**Fig. 9:**
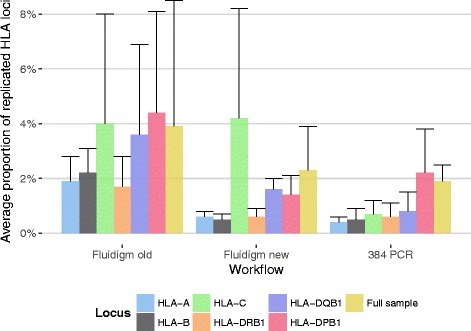
Monthly median repetition rate of HLA loci for different workflows (see Methods for details). Error bars show median absolute deviation (MAD). If more than 4 loci of a sample require repetition, the full sample (*yellow bars*) is repeated

**Fig. 10:**
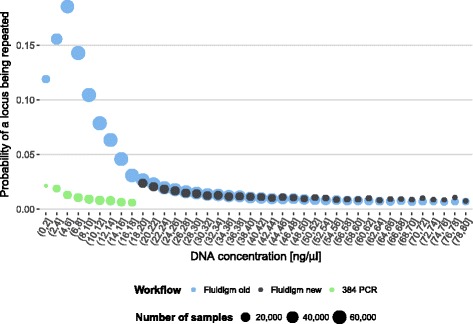
Dependency between initial template DNA concentration and the number of HLA loci per donor that have to be verified in repeat typing. Colours indicate different workflows (see Methods for detail). The point size is indicative of sample size

Between January 2013 and November 2015 (Fluidigm old workflow) a total of 3.6% of individual loci were repeated and a total of 4.4% of full samples were rerun. Both locus and workflow heavily affect repetition rates. Subjecting all samples to the Fluidigm workflow, HLA-C, -DQB1, and -DPB1 showed high average repetition rates around 4%, whilst HLA-A, -B, and -DRB1 showed average repetition rates around 2% (Fig. 9). The introduction of the 384 PCR workflow for low-concentration samples in November 2015 had a marked twofold effect on repetition rates. First, repetition rates decreased from 3.6 to 2.0% for individual loci and from 4.4 to 2.5% for full samples for the high-concentration samples still processed on Fluidigm chips. Such an improvement was expected since problematic samples with low DNA concentrations were shifted to another workflow. Second, and more unexpected, with 1.3% for individual loci and 2.5% for full samples repetition rates for the low-concentration samples now processed with the 384 PCR workflow were comparable to or lower than the repetition rates achieved for the high-concentration samples on the Fluidigm chips (Figs. 9 and 10). This effect is likely explained by the fact that low DNA concentrations on Fluidigm Access Array chips adversely affect allelic dropout rates [17]. Accordingly, we find that the likelihood of a locus being repeated correlates inversely to the initial template DNA concentration when using Fluidigm chips whilst this relationship is markedly less pronounced in the 384 PCR workflow (Fig. 10).

### Novel alleles

Our in-house genotyping software classifies and reports all consensus sequences differing from the reference alleles found in the IMGT/HLA database as potential novel alleles. Given the likelihood of such signatures being generated by artefacts such as PCR errors, co-amplification products, or noise, these samples need to be verified by repeat typing of an independent PCR product. Prior to 2015 this verification process was not performed automatically but was left to a technician’s discretion. As of January 2015, all potential novel sequences routinely undergo repeat typing. For this reason, we restrict the analysis described to samples typed between January 2015 and May 2016, for which data are exhaustive.

**Fig. 11:**
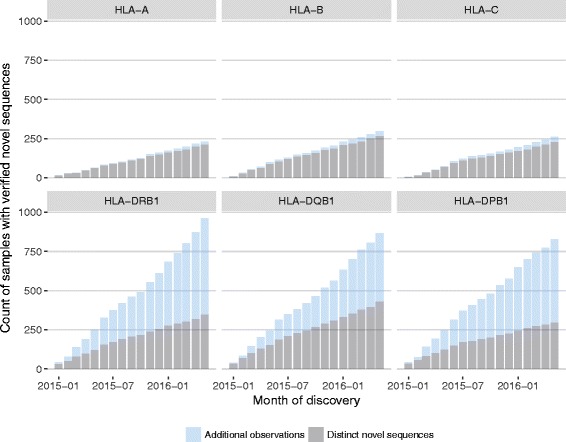
The cumulative numbers of novel HLA alleles discovered between January 2015 and May 2016 during routine genotyping of exons 2 and 3. All allelic sequences were verified by replicate typing using an independent PCR reaction. *Grey* shades denote distinct novel sequences; *blue* shades denote additional samples with previously observed novel sequences

During this period of 17 months, a total of 17,184,548 HLA alleles from 1,432,944 donors were typed. In the case of 13,835 (0.081%) alleles repeat typing was triggered because the signature of a potential novel allele was detected. 3,674 (26.4%) sequences could be confirmed as *bona fide* novel alleles, representing 1,919 unique novel sequences. In the remainder of the cases repeated sequencing suggested that the initial deviation from known alleles was caused by PCR or sequencing artefacts. It follows that we observe a maximum PCR error rate of 0.06%. A PCR error might mask a true novel allele if the error actually restored the polymorphism to the reference sequence. If we assume an average length of 730 bp for an allelic sequence composed of exons 2 and 3, we expect approximately one undetected novel allele in 5 million *bona fide* novel alleles. Novel alleles were not equally distributed amongst HLA loci. Overall we found class II loci three-fold over-represented amongst samples with novel alleles (2,816 class II loci vs. 858 class I loci; Fig. 11; Table 2). Interestingly, however, the rate of discovery of distinct novel sequences was far smaller amongst class II loci compared to class I loci (Class II: 40.5% unique novel sequence; Class I: 90.7% unique novel sequences; Fig. 11).

**Table 2:** Novel alleles discovered between January 2015 and May 2016

Locus	Samples	Total novel alleles	Distinct novel alleles	Estimates of number of distinct novel alleles at *n* typed samples		
				3 M	6 M	9 M
HLA-A	1.43 M	248	230	315	393	394
HLA-B	1.43 M	325	293	431	688	921
HLA-C	1.43 M	285	255	391	697	1001
HLA-DRB1	1.43 M	1011	364	702	1254	1806
HLA-DQB1	1.43 M	941	464	830	1443	2055
HLA-DPB1	1.43 M	864	313	615	1088	1561

Using these data we attempted to forecast the expected number of distinct novel alleles for the different HLA loci. First we resampled the original 1.43 million samples into 14 bins of 10^5^ samples each. This resampling procedure was repeated 30 times. Treating these bins as successive points in time (i.e., mimicking months of routine typing) we estimated the rate of discovery of distinct novel alleles at each of these successive sampling points. The expected decrease in discovery rate was modelled using a 3-parameter exponential decay model (Additional file 5: Figure S4). The models for the different loci were used to project the expected number of novel distinct alleles at 3, 6, and 9 million typed samples, respectively (Table 2).

It should be noted that these projections are subject to two important limitations: first they presume no change in the current ethnic make-up of donors processed at our lab (mainly Caucasians) and, second, they concern only novel alleles due to variation at exons 2 and 3 alone.

Given these restrictions, our estimates indicate that HLA-A is expected to be saturated for novel alleles (at exons 2 and 3) in the near future and that we can expect that most undiscovered variation is still harboured in class II loci, especially HLA-DQB1.

## Discussion

In recent years it has been extensively demonstrated that NGS can be applied to allele-level HLA typing with minimal ambiguity, providing benefits such as clonal sequencing to achieve in-phase reads and massive parallelism to enable the expansion of the regions sequenced [5, 6, 18–24]. Many tissue typing laboratories, however, have yet to fully embrace NGS technologies for routine high-throughput operations. DKMS Life Science Lab was one of the first labs to do so and has subsequently accumulated three and a half years of experience with routine HLA typing using an amplicon-based approach on the Illumina platforms. Over that period, more target genes were added (CCR5, ABO, RHD, and KIR) and genotyping data for 2.7 Mio samples was retrieved from 3,642 MiSeq and HiSeq sequencing runs (Fig. 1).

The amplicon-based approach was initially chosen for two major reasons. First, it is applicable to most genomic loci with modest modifications both in the laboratory workflow and the analysis software. This allowed rapidly supplementing the standard HLA typing portfolio with ABO, RHD, CCR5 and KIR typing as straightforward extensions to the core HLA typing workflow [8]. Perhaps equally important for production-scale operations is the simplicity and robustness of a PCR-based approach. Most steps in a PCR-based workflow can be automated using liquid handling robotic stations and high-throughput PCR solutions such as the Fluidigm Access Array System or high-capacity thermocyclers. Moreover, the amplicon-based approach proved highly resilient to the wide range of DNA quantities derived from the primary sources material (Fig. 2), tolerating as little as 2 ng/μl initial template DNA concentration (Fig. 7).

With our large-scale NGS HLA typing strategy we obtained high-resolution genotype assignments for an average of 98.9% of the HLA-A, -B, -C, -DRB1, -DQB1, and -DPB1 loci typed (Table 1). The lowest high-resolution typing rate was achieved for HLA-C (96.8%). HLA-C turned out to be a challenging locus in many regards: In the Fluidigm workflow, before the separation of low- and high-DNA-concentration samples, it showed the highest average primer dimer rate amongst all loci (32.8% ± 14.1% SD; Additional file 6: Figure S5) and, correspondingly, the lowest average numbers of on-target reads (Fig. 8). Exon 3 of HLA-C showed the highest proportion of detected chimeric reads amongst all amplicons (Fig. 6) and HLA-C is amongst the loci with the highest average repetition rates (Fig. 9).

However, many of these issues resolved or improved not only for HLA-C but also other loci when we started to treat low-DNA-concentration samples (<18 ng/μl) and high-DNA- concentration samples differently. Low initial DNA concentrations in conjunction with the nanolitre-scale reaction volumes on 192.24 Fluidigm Access Array chips and the time- and cost-effective single-PCR 4-primer approach caused problems that largely dissipated once standard microlitre reaction volumes and 2-primer PCR setups were used. Alternatively, nanofluidic chips could be used in combination with a conventional 2-primer chemistry, which would probably also resolve issues with primer dimers and PCR artefacts. It should also be noted that the sample repetition rates reported arise from two main cases: a) truly failed amplifications producing insufficient read depths for genotyping and b) results that are largely correct, but get flagged for repeat typing for verification purposes. This includes rare alleles as well as results that may have suffered from PCR drop out due to limited dilution. For low DNA concentration samples applied to the 192 chips, that effect, although still rare, becomes measurable [18]. Therefore, these repeats reflect our extremely high standards with regard to minimal error genotyping.

Whilst the Fluidigm workflow as originally devised [5] has some operational advantages, such as being less complex to automate and having low reagent costs, in our lab it remains a viable option only for high-quality samples. As the DNA concentration effects are relatively subtle, they often only become possible to track down after a large amount of data has accumulated. Thus, we now see clear evidence that sequential 2-primer PCRs should be preferred over 4-primer amplicon tagging workflows especially if increased formation of chimeric reads and primer dimers pose a risk for the intended application. We also suggest that proofreading polymerases should best be used in conjunction with PTO primers unless it is assured that exonuclease activity is effectively inhibited before heat activation. Finally, when low template DNA concentrations cannot be avoided for applications that are sensitive to stochastic dropout of alleles, a standard PCR setup might be a better fit than a nanofluidics platform.

Large-scale NGS-based typing of HLA in the context of donor registries with tens of thousands of samples processed each month requires substantial upfront investments in terms of laboratory automation, IT infrastructure and data analysis capacity. At DKMS Life Science Lab we currently employ 3 custom automatic DNA extraction platforms with a total capacity of more than 25,000 samples/week, 18 robotic liquid handlers, 3 hydrocyclers, 20 Fluidigm cyclers, and 3 HiSeq 2500 instruments for sequencing. On one HiSeq 2500 instrument, up to 10,000 samples can be jointly sequenced within 3 days using “Rapid Run Mode” and a dual flow cell run. Data analysis is performed using custom software [5], currently running on 320 Xeon processor cores and requiring 1.1 TB of storage capacity per month to retain FASTQ files and analysis data. However, for the more common use case of HLA typing in a clinical setting or a diagnostic lab, the requirements are typically much less demanding. Example workflows suitable for low- to medium-throughput HLA genotyping using NGS have been discussed extensively in the literature [4, 18, 20].

The next step in HLA genotyping subsequent to a NGS-based amplicon approach is promised by long-read single molecule sequencing technologies such as offered by sequencing systems by Pacific Biosciences (PacBio) or Oxford Nanopore Technologies (ONT). Combined with long-range PCR they hold the promise of efficient and accurate haplotype sequencing over multiple kilobases, thereby making available exon and intron sequences not covered by the current approach. At present the clinical impact of sequence information outside the peptide binding groove remains unknown except for variations encoding null alleles [25]. Once scalable HLA typing workflows and analysis software become available for these technologies, it would appear short-sighted to dismiss the clinical potential of fully characterised HLA alleles. However, whilst HLA typing is already feasible with PacBio instruments [26, 27], the limited benefit for donor search strategies seems not to justify the five-fold higher sequencing costs in the registry context. An alternative to PacBio-based systems may be provided by ONT’s high-throughput PromethION system which promises HiSeq-like throughput rates (https://www2.nanoporetech.com/products/specifications) but at the time of writing has not yet been released to customers.

## Conclusions

Although long-read sequencing platforms are likely to deliver the next revolution in HLA typing, they cannot at present truly compete in terms of throughput and costs per sample with the currently available widely applied and proven short-read-based approaches. We expect amplicon-based NGS typing workflows to remain, at least for the next few years, as the workhorse in high-volume targeted typing applications like HLA genotyping for donor registries. Our experience after genotyping more than 2.7 million samples confirms that the short-amplicon-based genotyping approach exhibits the robustness required for everyday routine high-throughput operation. In particular, compared to Sanger sequencing, the NGS based approach has proven to be by far more cost efficient, easier to handle and less error prone. Taking advantage of these lessons learned will help to further increase robustness and efficiency and raise awareness for potential pitfalls, thereby minimising spurious genotyping results. NGS has revolutionised whole genome sequencing and is starting to have a tremendous impact on targeted sequencing applications, enabling projects of a whole new scale and breadth.

## Methods

### Samples, DNA isolation and quantification

All donor samples analysed here were provided by DKMS and other bone marrow donor centres between January 2013 and June 2016. Primary source material was either whole blood (18%) or two self-administered buccal swabs per donor (82% of all samples). DNA was isolated from 150 μl whole blood or a single buccal swab using the magnetic-bead-based “chemagic DNA Blood Kit special” or “chemagic DNA Buccal Swab kit special” (Perkin Elmer, Baesweiler, Germany), respectively. DNA was eluted in 100 μl elution buffer (10 mM Tris-HCl pH8.0). DNA concentrations were measured by quantitative fluorescence staining (SYBR Green, Biozym, Hessisch Oldendorf, Germany) using the TECAN infinite 200Pro (Tecan, Männedorf, Switzerland) plate reader. Depending on DNA concentrations, typing profile and the time frame, distinct workflows were applied (see below). Samples with concentrations lower than 2 ng/μl were generally excluded from typing. Samples from DKMS-US have only been recently subject to genotyping at the DKMS Life Science Lab and were not included in analyses that compare samples of different provenance.

### Amplicon design

Primers were designed to target the core HLA exons 2 and 3 of HLA-A, -B, -C, -DRB1, -DQB1 and -DPB1 as described in Lange et al. [5]. Additional primers were designed to create amplicons spanning exons 2 and 3 to help resolve phase of class I loci. Phasing amplicon sizes range between 580 bp and 720 bp. ABO amplicons were designed for exons 6 and 7 as described in Lang et al. [8]. The blood group locus RHD was amplified for exon 5 and 6 in conjunction with the highly homologous gene RHCE as positive control for RHD negative samples. The CCR5 amplicon encloses a 32-bp deletion that confers high resistance against HIV-1 acquisition when homozygous [28]. For presence/absence typing of KIR genes, amplicons were designed for exons 4, 5 and 7. Amplicon sizes for HLA (excluding the phasing amplicons), blood groups and CCR5 range between 313 bp and 426 bp. PCR primer sequences will be provided to researchers upon request.

### PCR amplification

Until November 2015, all samples were processed on 48.48 Fluidigm Access Array chips (2,304 PCR reactions per chip) or on 192.24 Fluidigm Access Array chips (4,608 PCR reactions per chip; Fluidigm Corporation, South San Francisco, USA) using a single-PCR 4-primer protocol (detailed in Lange et al. [5]). These workflows are henceforth both referred to as *Fluidigm workflow*.

As of 2015-11-20, samples with DNA concentrations lower than 18 ng/μl have been processed with an alternative 2-PCR 2-primer workflow (henceforth referred to as *384 PCR workflow*). Samples with DNA concentrations higher than 18 ng/μl have continued to be processed with the Fluidigm workflow.

Briefly, for the Fluidigm workflow amplicons for HLA, ABO, RHD, and CCR5 are generated concurrently on Access Array chips. A single multiplexed PCR setup includes pairs of inner primers with target-specific 3’-tails and 5’-tails complementary to two outer primers carrying molecular identifier (MID) sequences and adaptors for sequencing on Illumina MiSeq or HiSeq 2500 instruments. The amplicons for KIR genes are generated separately on 384-well plates. The loading of the Access Arrays and the 384-well pipetting procedure are performed on TECAN Evo instruments (Tecan, Männedorf, Switzerland). The loading, thermocycling and harvesting of the Access Arrays takes approximately 5 h.

Samples with low-concentrated DNA have an increased risk of allelic dropout [17], increased primer dimer formation (this paper) and decreased average percentage of bases > Q30 across entire MiSeq/HiSeq runs (this paper). As of November 2015, we have therefore been applying a separate workflow (384 PCR) to samples with DNA concentrations <18 ng/μl. In this workflow all loci are amplified in 11 multiplexed primary PCR reactions in separate 384-well plates as detailed in Lang et al. [8] for ABO typing. Thermocycling is performed in hydrocyclers (LGC Genomics, Berlin, Germany) for approximately 2.5 h. Next, all amplicons of each sample are combined using volumes appropriate to maintain a balance of targeted amplicons using a CyBi-Well vario system (Analytik Jena AG, Jena, Germany). The CyBi-Well vario system is also used to setup a subsequent secondary PCR reaction to elongate the amplicons with MIDs and sequencing adapters for Illumina sequencing. Amplicon pooling, secondary PCR setup and thermocycling last about 1 h.

Whilst the Fluidigm workflow has remained technically the same since November 2015, the introduction of the 384 PCR workflow for low-concentration samples changed the average DNA-concentration of samples subjected to the Fluidigm workflow. To distinguish between these cases, we refer to the Fluidigm workflow before and after November 2015 as *Fluidigm old* and *Fluidigm new*, respectively.

### Amplicon pooling, purification and quantification

PCR products generated on the Access Array system are pooled for post-PCR processing. Several of these pools are subsequently combined in equimolar amounts based on qPCR quantification for sequencing on MiSeq or HiSeq 2500 instruments. Before August 2014, the DNA concentration after PCR was not normalised per sample prior to pooling for reasons of time efficiency and costs. Potentially, this can lead to severely unequal read distributions across samples. For the Fluidigm workflow, empirical analysis of the dependency of final read yield from initial DNA concentration allowed for introducing a novel equilibration strategy in August 2014 that effectively uses initial DNA concentrations for quantification-free post-PCR normalisation (Fig. 7).

Purification of several amplicon pools is then performed in parallel using SPRIselect Beads (Beckman Coulter, Brea, USA) with a ratio of 0.6:1 beads to DNA with a subsequent dilution step for quantification by qPCR. Pooling, purification and dilution for qPCR quantification are performed on Biomek instruments (Beckman Coulter, Brea, USA) within 70 min. For qPCR the Library Quant Illumina Kit (KAPA Biosystems, Boston, USA) with standards in a range from 0.2 fM to 20 pM was used on ECO Real-Time PCR cyclers (Illumina, San Diego, USA) or ABI-StepOnePlus qPCR cyclers (Thermo Fisher, Carlsbad, USA), respectively. Quantification lasts approximately 1 h. Barcoded amplicons are pooled separately for KIR and the other loci.

### Library preparation and MiSeq/HiSeq runs

The purified and quantified amplicon pools are mixed in equimolar amounts and prepared as recommended by Illumina (Illumina, San Diego, USA). Libraries are loaded at 6.5 pM to 8 pM onto MiSeq or HiSeq flow cells with 10% PhiX spiked in. Paired-end sequencing is performed at 249, 251 or 260 cycles, respectively. On average, library preparation takes 30 min for a MiSeq and approximately 60 min for a HiSeq library.

### Data analysis

All routine genotyping was performed using the in-house developed software neXtype as described elsewhere [5, 8]. For HLA and KIR typing the IMGT/HLA [29] and IPD/KIR [30] databases were used as references, respectively. For ABO and RHD typing a modified NCBI dbRBC database [31] was used as a reference. For CCR5 the sequence for the wildtype without deletion and a 32 bp deletion variant were retrieved from NCBI as reference. The rate of primer dimers is determined by computationally scanning for Illumina adaptor sequences in the forward or reverse reads.

## Additional files

Additional file 1: Figure S1. Prevalence of low-quality samples over time and for different sample provenances (DE, Germany; PL, Poland; UK, United Kingdom). (PDF 5 kb)
Additional file 2: Figure S2. Correlation between DNA concentrations derived from the first and second swab provided by donors. (PDF 408 kb)
Additional file 3: Table S1. Average monthly primer dimer rates split by workflows and sequencing instruments. (CSV 2 kb)
Additional file 4: Figure S3. Average primer dimer rates for different amplicons, genes, PCR setups and reaction volumes. PCR setups are a 1-PCR 4-primer setup vs. a standard 2-PCR 2-primer setup. Differences in reaction volumes are denoted by PCR (10 μl) and Fluidigm (10 nl). Only amplicons with an average PD rate > 0.2% are labelled in the plot. The y-axis is square root scaled to enhance readability. (PDF 5 kb)
Additional file 5: Figure S4. The decaying rate of discovery of distinct novel HLA alleles based on 1.43 Mio samples typed between January 2015 and May 2016. All novel alleles were discovered during routine genotyping of exons 2 and 3 and verified by replicate typing using an independent PCR reaction. Decrease in discovery rate was modelled using a 3-parameter exponential decay model. Grey shades denote distinct novel sequences; blue shades denote additional samples with previously observed novel sequences. See Results section for details. (PDF 7 kb)
Additional file 6: Figure S5. Mean primer dimer rates of HLA loci for different workflows. Error bars show standard deviation. (PDF 5 kb)

## Abbreviations

HLA: Human leucocyte antigen
HSCT: Haematopoietic stem-cell transplantation
NGS: Next generation sequencing
PCR: Polymerase chain reaction
PD: Primer dimer

## References

[1] Trowsdale J, Knight JC (2013). Major histocompatibility complex genomics and human disease. Annu Rev Genomics Hum Genet.

[2] Loiseau P, Busson M, Balere M-L, Dormoy A, Bignon J-D, Gagne K (2007). HLA association with hematopoietic stem cell transplantation outcome: the number of mismatches at HLA-A, -B, -C, -DRB1, or -DQB1 is strongly associated with overall survival. Biol Blood Marrow Transplant.

[3] Sauter J, Solloch UV, Giani AS, Hofmann JA, Schmidt AH (2016). Simulation shows that HLA-matched stem cell donors can remain unidentified in donor searches. Sci Rep.

[4] Grumbt B, Eck SH, Hinrichsen T, Hirv K (2013). Diagnostic applications of next generation sequencing in immunogenetics and molecular oncology. Transfus Med Hemotherapy.

[5] Lange V, Böhme I, Hofmann J, Lang K, Sauter J, Schöne B (2014). Cost-efficient high-throughput HLA typing by MiSeq amplicon sequencing. BMC Genomics.

[6] Wang C, Krishnakumar S, Wilhelmy J, Babrzadeh F, Stepanyan L, Su LF (2012). High-throughput, high-fidelity HLA genotyping with deep sequencing. Proc Natl Acad Sci.

[7] Zhou M, Gao D, Chai X, Liu J, Lan Z, Liu Q (2015). Application of high-throughput, high-resolution and cost-effective next generation sequencing-based large-scale HLA typing in donor registry. Tissue Antigens.

[8] Lang K, Wagner I, Schöne B, Schöfl G, Birkner K, Hofmann JA (2016). ABO allele-level frequency estimation based on population-scale genotyping by next generation sequencing. BMC Genomics BioMed Central.

[9] Rychlik W (1995). Selection of primers for polymerase chain reaction. Mol Biotechnol Humana Press.

[10] Chou Q, Russell M, Birch DE, Raymond J, Bloch W (1992). Prevention of pre-PCR mis-priming and primer dimerization improves low-copy-number amplifications. Nucleic Acids Res.

[11] Laver TW, Caswell RC, Moore KA, Poschmann J, Johnson MB, Owens MM (2016). Pitfalls of haplotype phasing from amplicon-based long-read sequencing. Sci Rep Nature Publishing Group.

[12] Meyerhans A, Vartanian J-P, Wain-Hobson S (1990). DNA recombination during PCR. Nucleic Acids Res.

[13] Lahr DJG, Katz LA (2009). Reducing the impact of PCR-mediated recombination in molecular evolution and environmental studies using a new-generation high-fidelity DNA polymerase. Biotechniques.

[14] Judo MS, Wedel AB, Wilson C (1998). Stimulation and suppression of PCR-mediated recombination. Nucleic Acids Res.

[15] Kanagawa T (2003). Bias and artifacts in multitemplate polymerase chain reactions (PCR). J Biosci Bioeng.

[16] Aird D, Ross MG, Chen W-S, Danielsson M, Fennell T, Russ C (2011). Analyzing and minimizing PCR amplification bias in Illumina sequencing libraries. Genome Biol BioMed Central.

[17] Schöfl G, Schmidt AH, Lange V (2016). Prediction of spurious HLA class II typing results using probabilistic classification. Hum Immunol.

[18] Danzer M, Niklas N, Stabentheiner S, Hofer K, Pröll J, Stückler C (2013). Rapid, scalable and highly automated HLA genotyping using next-generation sequencing: a transition from research to diagnostics. BMC Genomics.

[19] Bentley G, Higuchi R, Hoglund B, Goodridge D, Sayer D, Trachtenberg EA (2009). High-resolution, high-throughput HLA genotyping by next-generation sequencing. Tissue Antigens.

[20] Gabriel C, Fürst D, Faé I, Wenda S, Zollikofer C, Mytilineos J (2014). HLA typing by next-generation sequencing - getting closer to reality. Tissue Antigens.

[21] Lind C, Ferriola D, Mackiewicz K, Heron S, Rogers M, Slavich L (2010). Next-generation sequencing: the solution for high-resolution, unambiguous human leukocyte antigen typing. Hum Immunol.

[22] Erlich RL, Jia X, Anderson S, Banks E, Gao X, Carrington M (2011). Next-generation sequencing for HLA typing of class I loci. BMC Genomics.

[23] Hosomichi K, Shiina T, Tajima A, Inoue I (2015). The impact of next-generation sequencing technologies on HLA research. J Hum Genet Nature Publishing Group.

[24] Shiina T, Suzuki S, Ozaki Y, Taira H, Kikkawa E, Shigenari A (2012). Super high resolution for single molecule-sequence-based typing of classical HLA loci at the 8-digit level using next generation sequencers. Tissue Antigens.

[25] Elsner H-A, Blasczyk R (2004). Immunogenetics of HLA null alleles: implications for blood stem cell transplantation. Tissue Antigens.

[26] Mayor NP, Robinson J, McWhinnie AJM, Ranade S, Eng K, Midwinter W (2015). HLA typing for the next generation. PLoS One Public Library of Science.

[27] Chang C-J, Chen P-L, Yang W-S, Chao K-M (2014). A fault-tolerant method for HLA typing with PacBio data. BMC Bioinformatics.

[28] Hütter G, Nowak D, Mossner M, Ganepola S, Müßig A, Allers K (2009). Long-Term Control of HIV by CCR5 Delta32/Delta32 Stem-Cell Transplantation. N Engl J Med Massachusetts Medical Society.

[29] Robinson J, Mistry K, McWilliam H, Lopez R, Parham P, Marsh SGE (2011). The IMGT/HLA database. Nucleic Acids Res.

[30] Robinson J, Mistry K, McWilliam H, Lopez R, Marsh SGE (2010). IPD--the immuno polymorphism database. Nucleic Acids Res.

[31] Patnaik SK, Helmberg W, Blumenfeld OO (2012). BGMUT: NCBI dbRBC database of allelic variations of genes encoding antigens of blood group systems. Nucleic Acids Res.

