# Supplementary figures and images for: 2.7 million samples genotyped for HLA by next generation sequencing: lessons learned
Source: BMC Genomics. 2017 Feb 14;18:161. doi: 10.1186/s12864-017-3575-z (PMC5309984; doi:10.1186/s12864-017-3575-z)

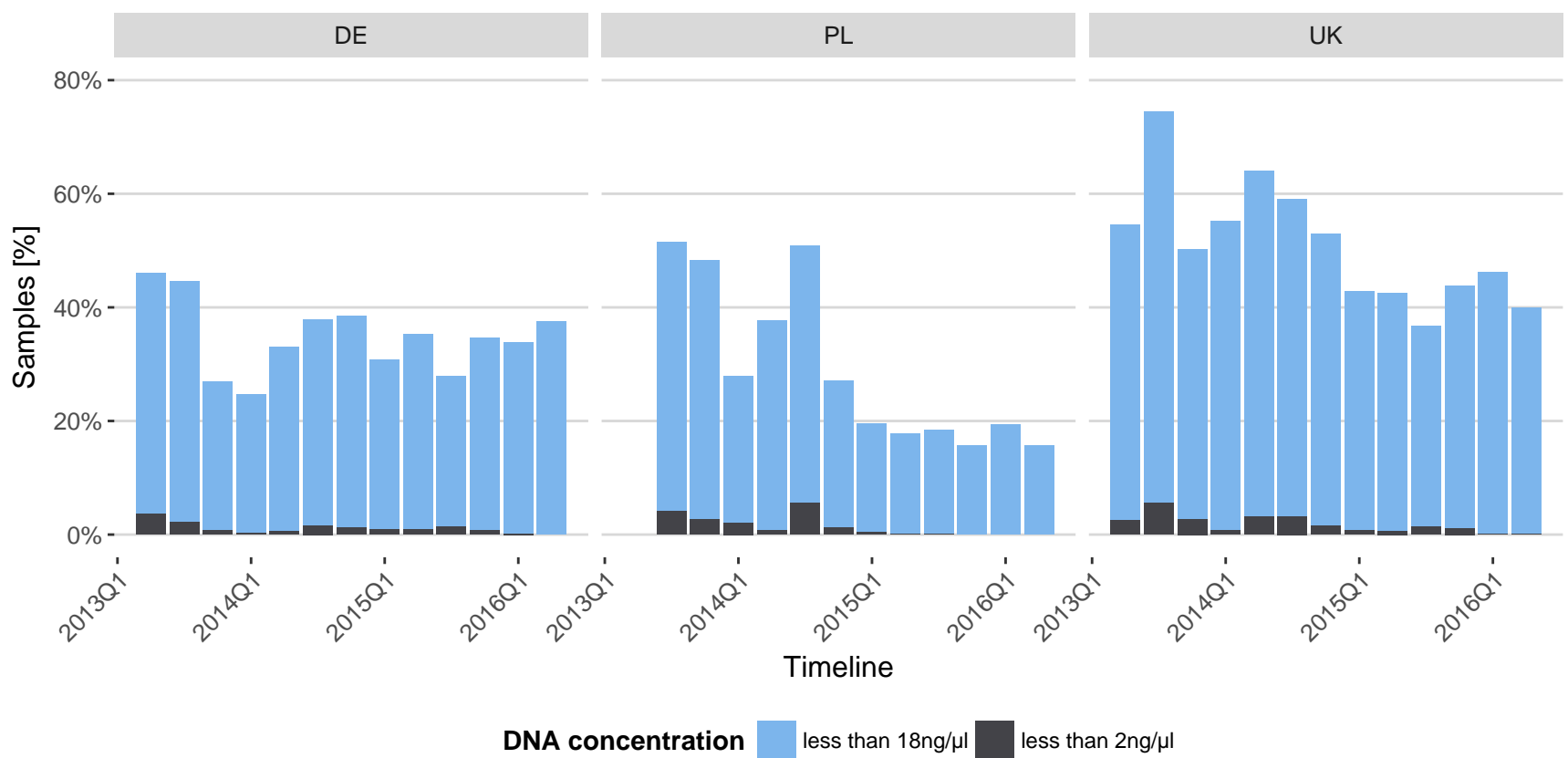

Supplement: Additional file 1: Figure S1. — Prevalence of low-quality samples over time and for different sample provenances (DE, Germany; PL, Poland; UK, United Kingdom). (PDF 5 kb) [file 12864_2017_3575_MOESM1_ESM.pdf]

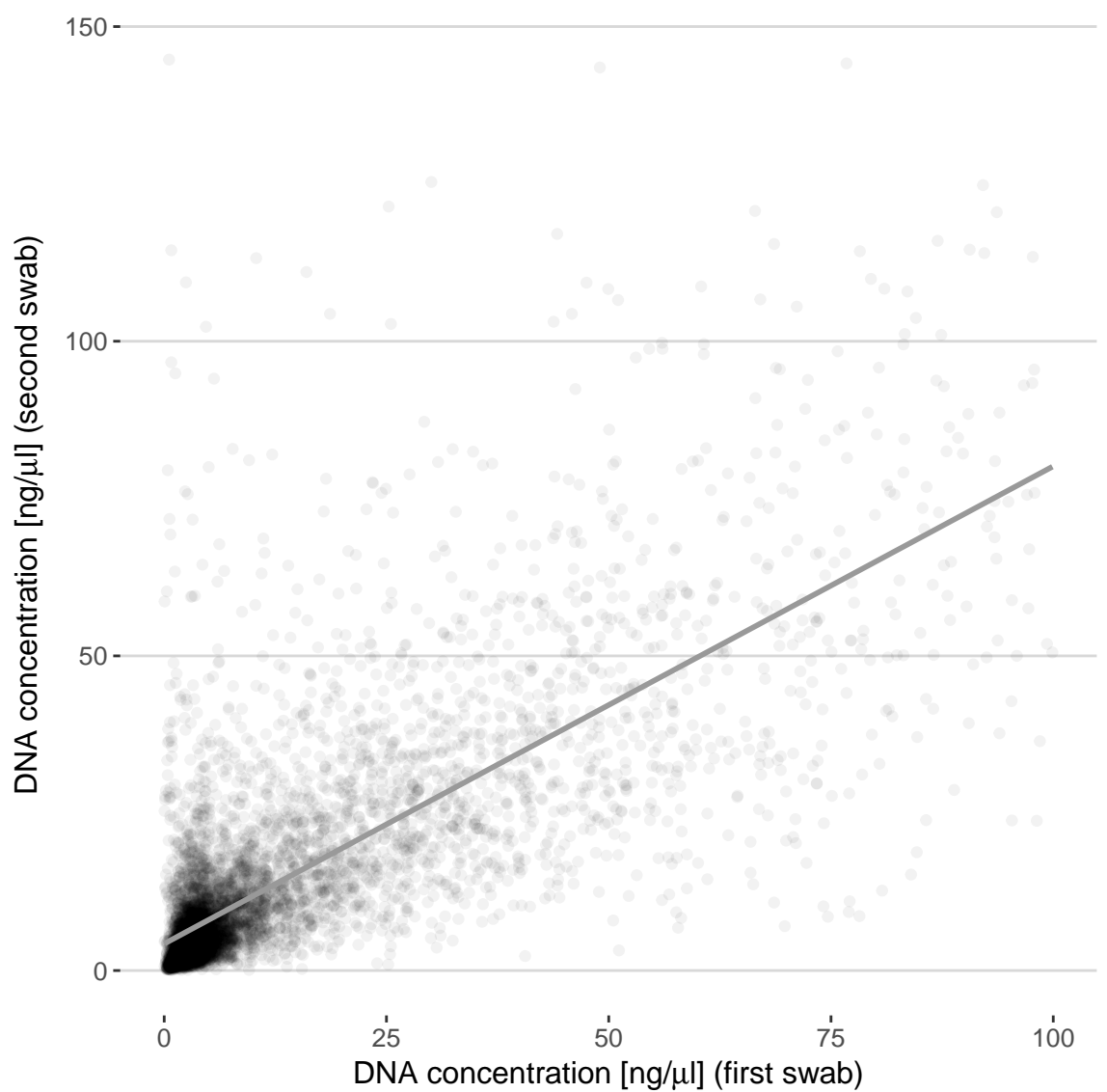

Supplement: Additional file 2: Figure S2. — Correlation between DNA concentrations derived from the first and second swab provided by donors. (PDF 408 kb) [file 12864_2017_3575_MOESM2_ESM.pdf]

Mean PD rate

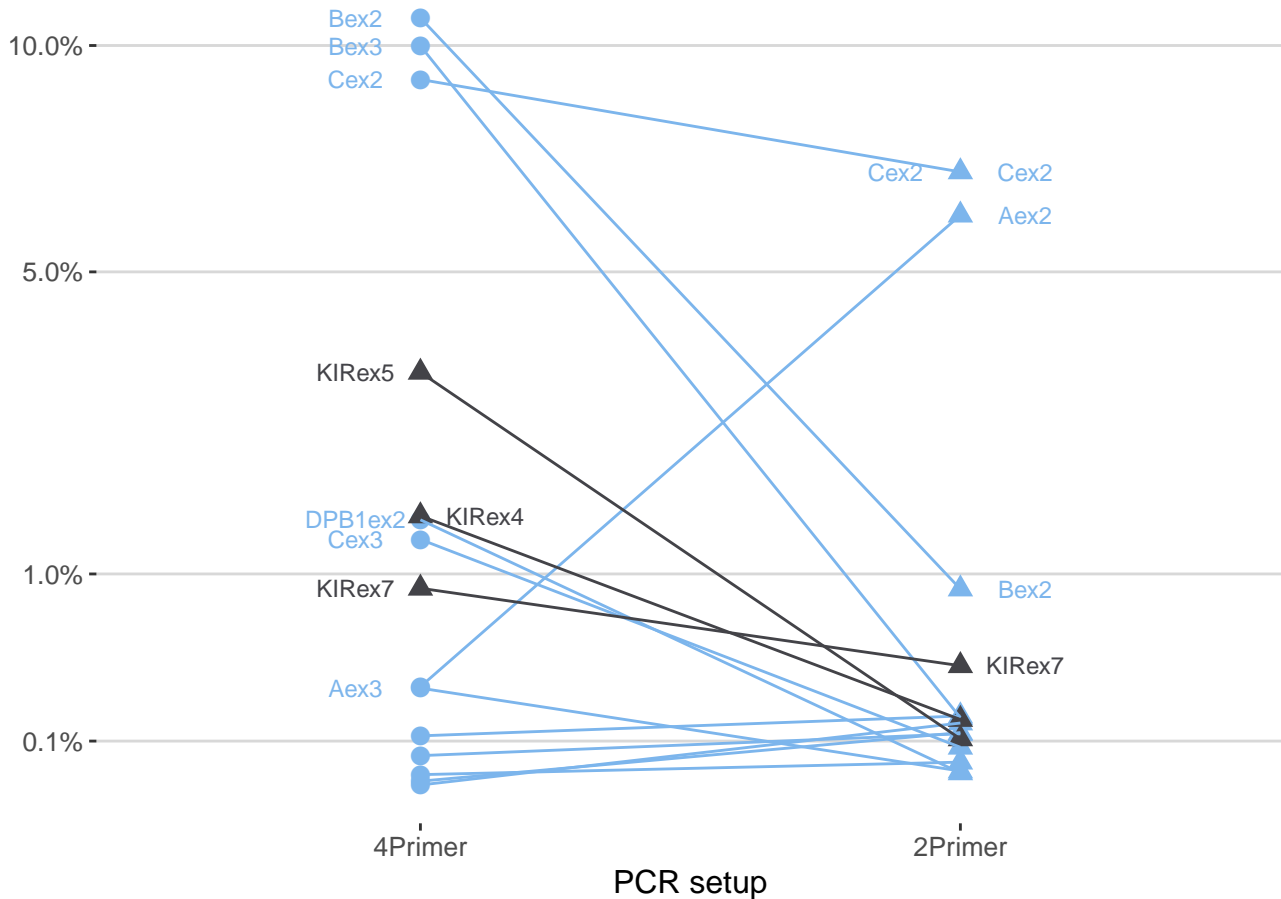

Gene ● HLA ● KIR Volume ● Fluidigm ▲ PCR

Supplement: Additional file 4: Figure S3. — Average primer dimer rates for different amplicons, genes, PCR setups and reaction volumes. PCR setups are a 1-PCR 4-primer setup vs. a standard 2-PCR 2-primer setup. Differences in reaction volumes are denoted by PCR (10 μl) and Fluidigm (10 nl). Only amplicons with an average PD rate > 0.2% are labelled in the plot. The y-axis is square root scaled to enhance readability. (PDF 5 kb) [file 12864_2017_3575_MOESM4_ESM.pdf]

Average primer dimer rate

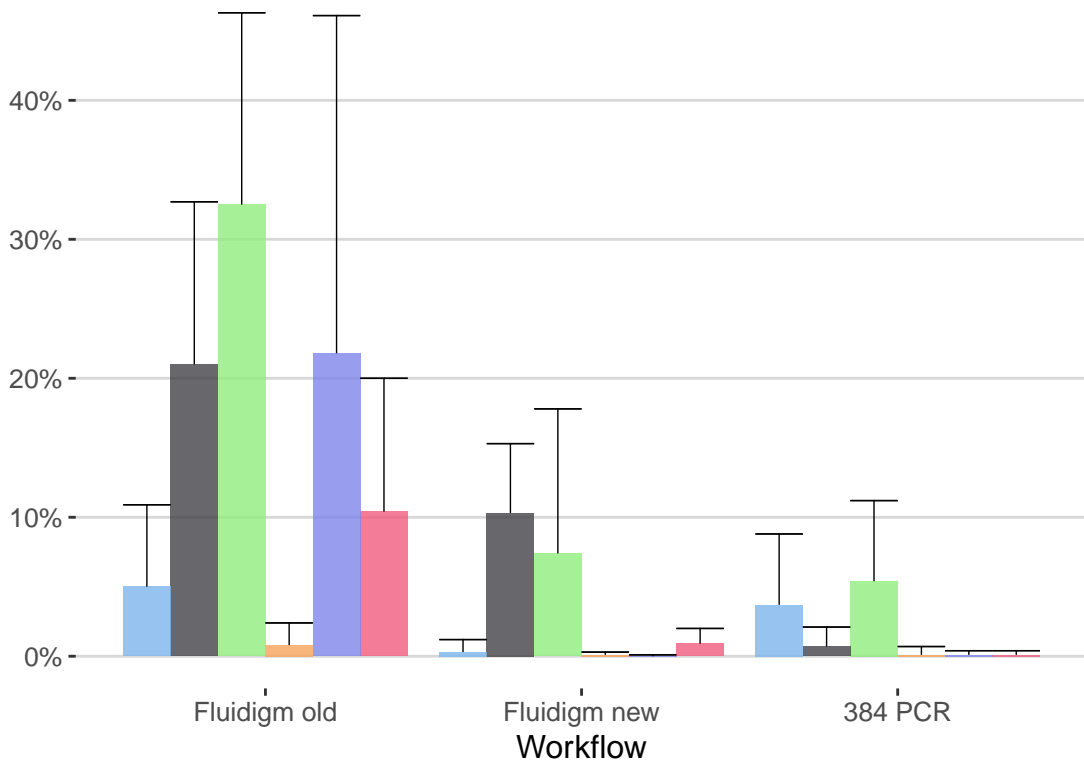

Locus

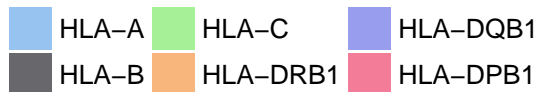

Supplement: Additional file 6: Figure S5. — Mean primer dimer rates of HLA loci for different workflows. Error bars show standard deviation. (PDF 5 kb) [file 12864_2017_3575_MOESM6_ESM.pdf]
