# Supplementary material for: 2.7 million samples genotyped for HLA by next generation sequencing: lessons learned
Source: BMC Genomics. 2017 Feb 14;18:161. doi: 10.1186/s12864-017-3575-z (PMC5309984; doi:10.1186/s12864-017-3575-z)
Supplement: Additional file 5: Figure S4. — The decaying rate of discovery of distinct novel HLA alleles based on 1.43 Mio samples typed between January 2015 and May 2016. All novel alleles were discovered during routine genotyping of exons 2 and 3 and verified by replicate typing using an independent PCR reaction. Decrease in discovery rate was modelled using a 3-parameter exponential decay model. Grey shades denote distinct novel sequences; blue shades denote additional samples with previously observed novel sequences. See Results section for details. (PDF 7 kb) [file 12864_2017_3575_MOESM5_ESM.pdf]

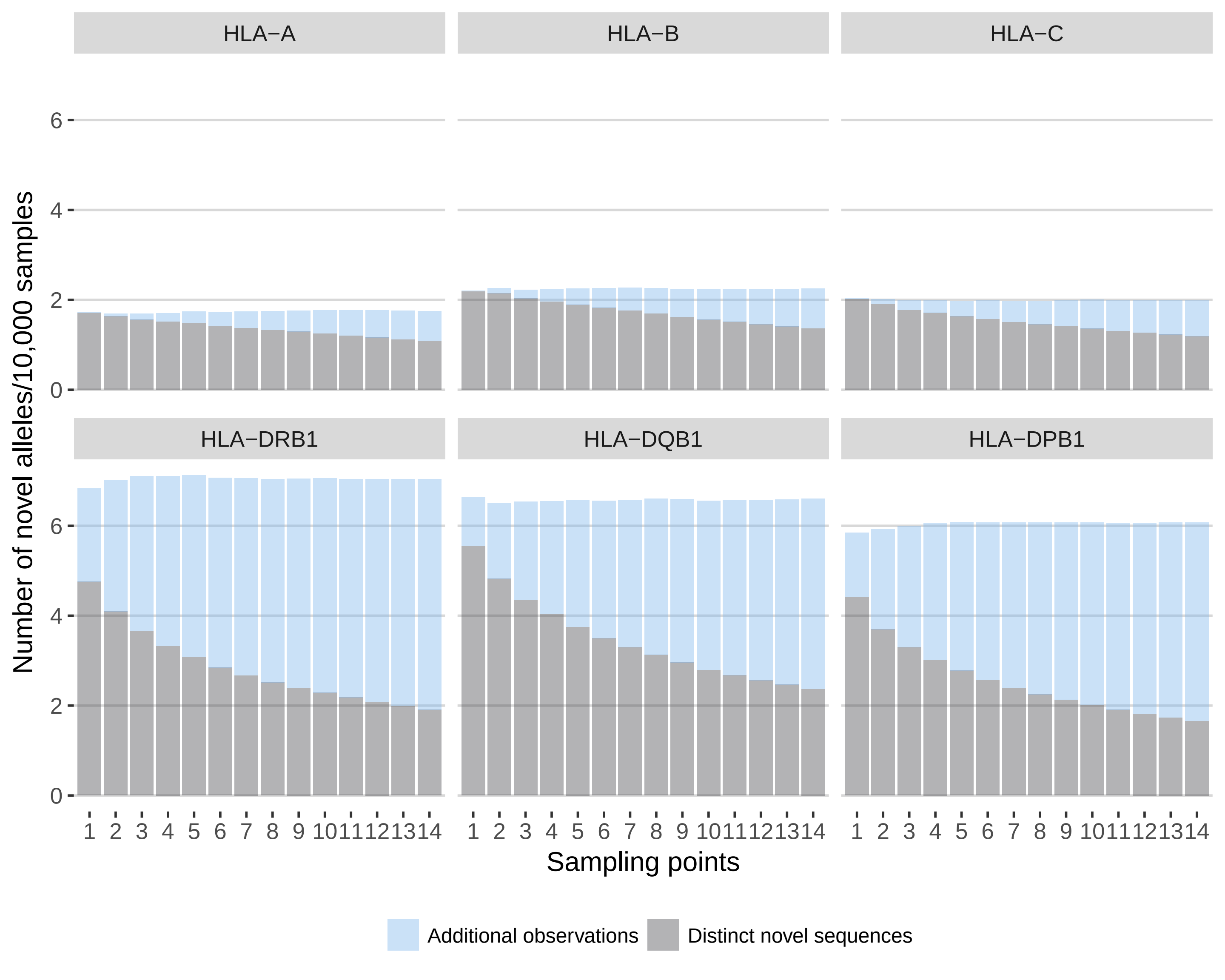
HLA−A
HLA−B
HLA−C
HLA−DRB1
HLA−DQB1
HLA−DPB1
0
2
4
6
1 2 3 4 5 6 7 8 9 10 11 12 13 14
Number of novel alleles/10,000 samples
Sampling points
Additional observations
Distinct novel sequences
